# Bioanalytics for Influenza Virus-Like Particle Characterization and Process Monitoring

**DOI:** 10.3389/fbioe.2022.805176

**Published:** 2022-02-18

**Authors:** Sofia B. Carvalho, Ricardo J. S. Silva, Marcos F. Q. Sousa, Cristina Peixoto, António Roldão, Manuel J. T. Carrondo, Paula M. Alves

**Affiliations:** ^1^ iBET, Instituto de Biologia Experimental e Tecnológica, Oeiras, Portugal; ^2^ Instituto de Tecnologia Química e Biológica António Xavier, Universidade Nova de Lisboa, Oeiras, Portugal

**Keywords:** influenza, vaccines, virus-like particles, analytical tools, biophysical characterization, biochemical characterization, bioprocess monitoring

## Abstract

Virus-like particles (VLPs) are excellent platforms for the development of influenza vaccine candidates. Nonetheless, their characterization is challenging due to VLPs’ unique biophysical and biochemical properties. To cope with such complexity, multiple analytical techniques have been developed to date (e.g., single-particle analysis, thermal stability, or quantification assays), most of which are rarely used or have been successfully demonstrated for being applicable for virus particle characterization. In this study, several biophysical and biochemical methods have been evaluated for thorough characterization of monovalent and pentavalent influenza VLPs from diverse groups (A and B) and subtypes (H1 and H3) produced in insect cells using the baculovirus expression vector system (IC-BEVS). Particle size distribution and purity profiles were monitored during the purification process using two complementary technologies — nanoparticle tracking analysis (NTA) and tunable resistive pulse sensing (TRPS). VLP surface charge at the selected process pH was also assessed by this last technique. The morphology of the VLP (size, shape, and presence of hemagglutinin spikes) was evaluated using transmission electron microscopy. Circular dichroism was used to assess VLPs’ thermal stability. Total protein, DNA, and baculovirus content were also assessed. All VLPs analyzed exhibited similar size ranges (90–115 nm for NTA and 129–141 nm for TRPS), surface charges (average of −20.4 mV), and morphology (pleomorphic particles resembling influenza virus) exhibiting the presence of HA molecules (spikes) uniformly displayed on M1 protein scaffold. Our data shows that HA titers and purification efficiency in terms of impurity removal and thermal stability were observed to be particle dependent. This study shows robustness and generic applicability of the tools and methods evaluated, independent of VLP valency and group/subtype. Thus, they are most valuable to assist process development and enhance product characterization.

## Introduction

Influenza VLPs are traditionally composed of hemaglutinin (HA) protein displayed on the surface of M1 protein (the scaffold). They can be composed by HA of only one subtype/strain (i.e., monovalent) or multiple subtypes/strains (i.e., multivalent). These VLPs are enveloped particles, thus they contain host cell proteins (HCP) acquired during the budding process (Vicente et al., 2011). These particles can be produced using several expression systems, namely, *Escherichia coli*, eukaryotic mammalian, insect, and plant cells (Pillet et al., 2016; Tretyakova et al., 2016; Venereo-Sanchez et al., 2016; Huang et al., 2017). The presence of HCP is highly dependent on the expression system used (Venereo-Sanchez et al., 2016). The envelope composition affects particle complexity impacting VLPs’ structure and stability (Deng, 2018; González-Domínguez et al., 2020).

The integrity and stability of the VLP, including detection of all HA proteins, should be monitored during the entire bioprocess as VLP degradation can occur at any point during the process. There is a need for a deeper characterization of influenza VLPs and their bioprocess, to provide us more insights on their components and product- and process-related impurities critical for improving manufacturing. However, traditional methods used for influenza viruses are not enough to cope with the VLPs’ characterization needs (Thompson et al., 2013; Nooraei et al., 2021). Furthermore, the implementation of several of these methods for influenza VLPs and their optimization for in-process samples and different expression systems is yet not fully accomplished.

Recently, new analytical tools are being developed and efforts are being made to revise methods used in more mature fields or to characterize other types of products (e.g., monoclonal antibodies). A comprehensive toolbox of analytical tools, traditional and modern ones, for the structural, functional, and potency characterization of VLPs was reviewed by Huang et al. (2017) and Nooraei et al. (2021) and are summarized in Table 1. The biochemical characterization should consider the determination of VLPs’ purity, global charge, and relevant proteins’, such as HA and M1, primary amino acid sequence, and molecular mass. In biophysical analysis, parameters such as morphology, size, polydispersity, thermal stability, and aggregation propensity are evaluated. Immunoassays tools have been used for the biological characterization of VLPs by analyzing the binding of functional epitopes of the particles to a panel of specific monoclonal antibodies.

**TABLE 1:** Bioanalytics for VLP characterization. Summary of analytical tools used for biochemical, biophysical and biological characterization of VLPs. Reviewed in Huang et al. (2017) and Nooraei et al. (2021).

Biochemical characterization			
Amino acid sequence	Molecular weight	Purity	Isoelectric point
LC-MS (peptide mapping)	SDS-PAGE^a^; WB^a^; MALDI-TOF MS nativeMS	RP-HPLC; SDS-PAGE^a^	icIEF
Biophysical characterization			
Morphology	Size and polydispersity	Stability and aggregation propensity	Particle concentration
TEM^a^; cryo-EM; AFM	TEM; cryo-EM; AFM; TRPS^a^; DLS; NTA^a^; AUC; AF4-MALS	CD^a^; DSC; Cloud point	TRPS^a^; DLS; NTA^a^
Biological characterization			
Antibody binding	Antigenicity	Receptor binding	
SPR; BLI	ELISA	Hemagglutination assay	

LC-MS, liquid chromatography-mass spectrometry; SDS-PAGE, sodium dodecyl sulfate–polyacrylamide gel electrophoresis; WB, western blot; MALDI-TOF: matrix-assisted laser desorption/ionization–Time of Flight; RP-HPLC, reversed-phase–high-performance liquid chromatography; icIEF, imaged capillary isoelectric focusing; TEM, transmission electron microscopy; cryo-EM, cryogenic electron microscopy; AFM, atomic force microscopy; TRPS, tunable resistive pulse sensing; DLS, dynamic light scattering; NTA, nanoparticle tracking analysis; AUC, analytical ultracentrifugation; AF4-MALS, Asymmetrical flow field-flow fractionation—multi angle light scattering; CD, circular dichroism; DSC, differential scanning calorimetry; SPR, surface plasmon resonance; BLI, biolayer interferometry analysis; ELISA, enzyme-linked immunosorbent assay.

a Explored in this work.

Besides HCP, there are other product-related impurities such as extracellular vesicles (EVs), described for example for HEK293 system (Venereo-Sanchez et al., 2016; Moleirinho et al., 2019), and/or baculoviruses (BV) when using the insect cell system (Margine et al., 2012; Carvalho et al., 2019). Although host cell-derived proteins and particles such as EVs or BV can play a role in the immunological response, they can also pose regulatory issues and should be monitored during the bioprocess (Margine et al., 2012; Thompson et al., 2015; Deng, 2018; Lavado-García et al., 2020). The discrimination of VLPs from impurities such as EVs or BV, which share similar surface properties, requires the use of orthogonal tools for both analytical and purification tasks. Recent advances to these tools are reported elsewhere (Thompson et al., 2013; Carvalho et al., 2016; Moleirinho et al., 2020; Reiter et al., 2020).

In the current work, a set of six different influenza VLPs was expressed using the insect cells — recombinant baculovirus expression vector system (IC-BEVS), exploring distinct valences (i.e. mono- and pentavalent), subtypes (i.e. H1 and H3), and groups (A and B). Biochemical (HA titer and impurities, i.e. total protein, DNA, and baculovirus content) and biophysical (VLP identity, morphology, size distribution, surface charge, structure and stability) analyses were performed using different methods. The main goal was to demonstrate the applicability of the analytical tools used and how they can be applied to bioprocess monitoring and to guide its optimization.

## Materials and Methods

### Cell Line and Culture Media

Insect High Five cells (B85502, ThermoFisher Scientific) were routinely sub-cultured in 500 ml shake flasks (10% working volume) (Corning) to 0.3 × 10^6^ cells/ml inoculum, every 3–4 days, when cell concentration reached 2–3×10^6^ cells/ml, using Insect X-press medium (BEBP12-730Q, Sartorius). An Innova 44R incubator (New Brunswick) at 27°C and 100 rpm (orbital motion diameter of 2.54 cm) was used.

### Baculovirus Amplification

Recombinant baculoviruses containing influenza matrix M1 gene in combination with one or multiple hemagglutinin (HA) genes were generated by RedBiotech AG (Switzerland) using its proprietary rePAX^®^ technology (Supplementary Table S1). The promotor used for both HA and M1 was the polh (polyhedrin promoter). HA and M1 protein sequences are described in Supplementary Table S2. Amplification of baculovirus stocks was performed as described elsewhere (Vieira et al., 2005). Briefly, Spodoptera frugiperda Sf-9 cells (11496-015, ThermoFisher Scientific) were infected at 1 × 10^6^ cells/ml at a multiplicity of infection (MOI) of 0.1 infectious particles per cell (IP/cell). When cell viability reached 80%–85%, culture bulk was harvested and centrifuged at 200×*g* for 10 min at 4°C. The pellet was discarded, and the supernatant was centrifuged at 2,000×g for 20 min at 4°C. The resulting supernatant was stored at 4°C until further use.

### Production of Influenza VLPs

Influenza VLPs were produced in shake flasks (2 L with 10% working volume). Briefly, shake flask cultures were infected at a cell concentration at infection (CCI) of 2 × 10^6^ cells/ml using an MOI of 1 IP/cell. Cell cultures were harvested when cell viability dropped to values around 40%, commonly between 48 h post-infection (hpi) and 72 hpi.

### Purification of Influenza VLPs

Influenza VLP purification started with the clarification process that was performed by sequential depth filtration using a D0HC filter (MD0HC23CL3, Merck Millipore) and an Opticap XL150 capsule with 0.5/0.2 µm pore size (KHGES015FF3, Merck Millipore), connected in series. The second step included an anion exchange chromatography by using a Sartobind Q MA 75 (93IEXQ42DB-12V, Sartorius) membrane adsorber, operated in bind/elute mode. The membrane adsorber was equilibrated with 50 mM HEPES, pH 7.0, and 150 mM of NaCl equilibration buffer. The flow rate was set to 5 membrane volume (MV) min^−1^ and the VLPs were collected in the elution step that was performed with 50 mM HEPES, pH 7.0, 1 M NaCl. The Sartobind Q elution pool containing VLPs was concentrated by tangential flow filtration (TFF) conducted using a flat sheet Pellicon XL Ultrafiltration Module Biomax 300 kDa 0.005 m^2^ (PXB300C50, Merck Millipore). The membrane module was set up according to the manufacturer’s instructions: it was preconditioned with deionized water, to eliminate trace preservatives and equilibrated with 50 mM HEPES, pH 7.0, and 200 mM NaCl (working buffer) before the concentration step. After achieving the desired concentration factor, the TFF loop was completely drained and the VLP retentate was recovered. Ultrafiltration retentate was loaded into a size-exclusion chromatography (SEC) performed using a HiLoad 26/600 Superdex 200 pg column (Cytiva). The column was loaded at a constant flow rate of 5 ml/min. Working buffer was used as eluent and the eluted fractions were collected for further analysis. The elution of influenza VLPs was monitored at 280 nm. The fraction containing the top of the chromatographic peak was collected and considered for further analysis (final). Both Sartobind Q and SEC chromatographies were coupled to an AKTA explorer 150 liquid chromatography system (Cytiva) equipped with UV, conductivity, and pH monitors. System operation and data gathering and analysis were performed using the UNICORN 6.0 software (Cytiva). All purification steps were performed at room temperature (RT) (22°C).

### Analytics

#### Cell Concentration and Viability

Cell concentration and viability were monitored daily using Cedex High-Resolution Cell Analyzer (Roche) or the trypan blue exclusion dye method with a Fuchs–Rosenthal hemocytometer.

#### Hemagglutination Assay

HA protein content was determined using the hemagglutination assay as described elsewhere (Carvalho et al., 2018; Sequeira et al., 2018). Briefly, samples were serially diluted 1:2 or 1:3 with DPBS(−/−) 1X (14190-169, Gibco) in V-bottom 96-well plates (Thermo Scientific) and gently mixed 1:1 with 1% chicken erythrocytes (Lohmann Tierzucht GmbH). An influenza vaccine (Influvac, Abbott) was used as a positive control, with known concentration. Plates were incubated at 4°C for at least 30 min. Hemagglutination was inspected visually and HA titer was estimated as being the inverse of the highest dilution of the sample that completely inhibited hemagglutination.

#### Turbidity

To evaluate the efficacy of the clarification step, turbidity of the harvest and clarified samples was measured using a Turbidimeter (2100 Qis Portable HACH).

#### Tunable Resistive Pulse Sensing

VLP size distribution, particle concentration, and charge (zeta potential) were evaluated using TRPS. These measurements were performed with the qNano (Izon Sciences) equipment and nanopore membranes NP200 (Izon Sciences), rated for particles between 80 and 500 nm. The instrument was set up and calibrated according to the manufacturer’s instructions. To calibrate size and particle concentration, carboxylated polystyrene particles with a mode diameter of 340 nm (SKP400E, Izon Science) with a concentration of 6 × 10^10^ particles/ml were used. All measurements were done using a membrane stretch of 45 mm, a voltage of 0.20 V, and a pressure of 5 mbar. The measurement duration was dependent on the number of particles detected with a minimum of 500 events. Zeta potential measurements were performed using PBS buffer using calibration particles with a diameter of 220 nm (CPC 200, Izon Science) and a nanopore membrane NP200 (Izon Science). The selected membrane stretch was 45.30 mm. The pressure (P) and voltage (V) calibration points were selected as V1P1, V1P2, V2P1, and V3P1 with V1, V2, and V3 as 0.32, 0.26, and 0.29 V, respectively, and P1 and P2 as 1 and 2 mbar. The zeta potential of VLP samples was measured using V1P1 voltage and pressure setting.

#### Transmission Electron Microscopy

TEM was used to assess the integrity and morphology of the VLPs after downstream processing (ultrafiltrated retentate and selected SEC fractions). Sample preparation was performed as follows: a drop (5 µl) of each sample was adsorbed onto a formvar coated 150 mesh copper grid from Veco (Science Services) for 2 min. After washing the grid five times with sterile filtered dH_2_O, it was soaked in 2% uranyl acetate for 2 min and dried in air at RT (22°C). A Hitachi H-7650 120 kV transmission electron microscope (Hitachi High-Technologies Corporation) was used to analyze the samples.

#### Nanoparticle Tracking Analysis

VLP concentration and size distribution was measured using the NanoSight NS500 (Nanosight Ltd). Samples were diluted in D-PBS (14190-169, Gibco) to a particle concentration between 10^8^ and 10^9^ particles/ml to work at the instrument’s linear range. All measurements were performed at a controlled temperature of 25°C. Sample videos were analyzed with the Nanoparticle Tracking Analysis (NTA) 3.3 Analytical software. Capture settings (shutter and gain) were adjusted manually for each analysis. For each sample, five captures of 60-s videos were acquired and the total number of particles was considered.

#### Total Protein Quantification

Total protein content in samples was quantified using the BCA Protein Assay Kit (23225, Thermo Fisher Scientific) following the manufacturer’s instructions. Bovine serum albumin (BSA) was used for calibration (23209, Thermo Fisher Scientific). Absorbance at 562 nm was measured on Infinite M200 PRO NanoQuant (Tecan) microplate multimode reader using a clear 96-well microplate (655101, Greiner Bio-One GmbH).

#### Total dsDNA Quantification

Total dsDNA was determined using the Quant-iT Picogreen dsDNA reagent (P7581, Molecular Probes) according to the manufacturer’s instructions. Fluorescence (*λ*_exc_ = 485 nm, *λ*_emiss_ = 535 nm) was measured on an Infinite 200 PRO NanoQuant (Tecan) microplate multimode reader using a black 96-well microplate, flat transparent (3915, Corning).

#### SDS-PAGE and Western Blot Analysis

Influenza M1 and HA proteins in samples before and after purification were identified by SDS-PAGE and Western blot analysis. Samples were incubated at 70°C for 10 min after the addition of loading buffer (LDS sample buffer and reducing agent; ThermoFisher Scientific). Denatured samples were loaded into a 4%–12% NuPAGE Bis-Tris protein gel (ThermoFisher Scientific) using the MOPS running buffer (ThermoFisher Scientific). Loading was performed using 2 µg of total protein per lane and 0.04 µg of HA per lane. SeeBlue Plus2 Prestained Standard (ThermoFisher Scientific) molecular weight markers were used. Samples were resolved for 60 min at a constant voltage (200 V and 400 mA) and transferred to a nitrocellulose membrane using the iBlot system (ThermoFisher Scientific). SDS-PAGE and Western blot membranes were used in duplicate for M1 and HA protein identification. Membranes were blocked for 1 h with 5% (w/v) of dry milk (Merck Millipore) in Tris-buffered saline with 0.1% (w/v) of Tween 20 (T-TBS buffer). After blocking, membranes were incubated overnight at room temperature with the respective primary antibodies (Supplementary Table S3).

Western blot detection was performed with the corresponding anti-goat, anti-mouse, or anti-sheep secondary antibody conjugated with alkaline phosphatase for M1 and HA identification (Supplementary Table S3). Protein band detection was performed by covering membranes with NBT/BCIP 1-Step (Thermo Scientific) for 10 min; membranes were then scanned with a benchtop scanner device.

#### Baculovirus Quantification

Baculovirus DNA was extracted and purified using the High Pure Viral Nucleic Acid Kit (Roche Diagnostics) following the manufacturer’s instructions. The number of genome copies was quantified by real-time quantitative PCR (qPCR) following the protocol described elsewhere (Vicente et al., 2009). The master mix was prepared using the Light Cycler 480 SYBR Green I Master (04707516001, Roche Diagnostics), a final concentration of 0.5 µM of each primer, for the ie-1 baculovirus gene region and PCR grade water; 96-well white plates (04729692001, Roche Diagnostics) and a LightCycler 480 Instrument II (Roche Molecular Systems, Inc.) were used.

#### Circular Dichroism Spectroscopy

CD experiments were performed using a Chirascan™ qCD spectrometer (Applied Photophysics). Far UV CD spectra (*n* = 3) were measured at 20°C with a bandwidth of 1 nm and a time per point of 3 s from 195 to 260 nm. Temperature ramps (*n* = 1) were recorded by increasing the temperature from 20°C to 95°C from 195 to 260 nm, with a time per point of 0.4 s and spectra acquisition every 1°C. CD spectra at 90°C and 20°C after temperature ramp were also acquired. The HA concentration was 4.5 μg/ml. Temperature ramp melting temperature (Tm) was interpolated applying a sigmoidal regression performed using GraphPad version 9.1.1.

## Results

### Production of Influenza VLPs

**FIGURE 1:**
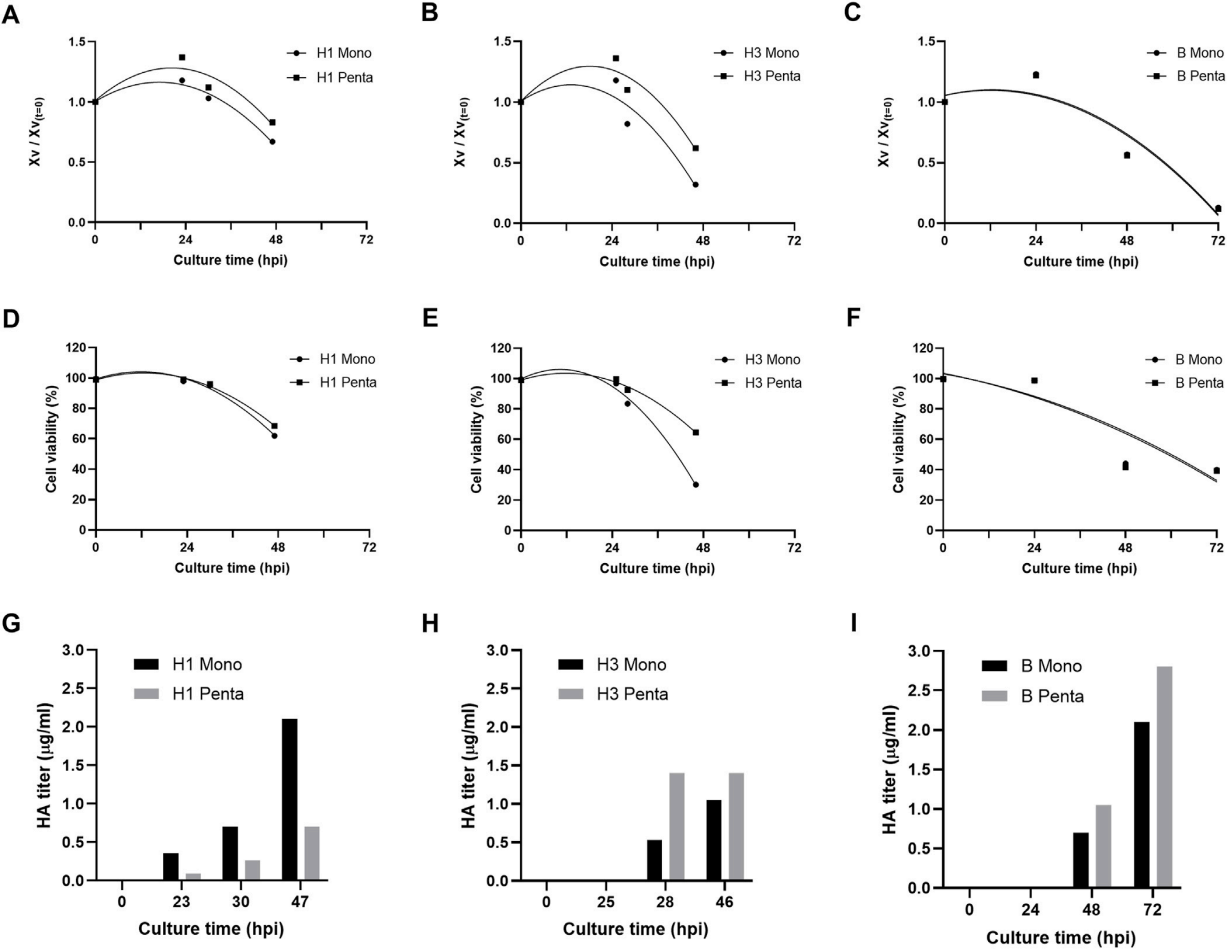
Production of influenza VLPs. **(A–C)** Normalized viable cell concentration during culture time upon infection, hours post-infection (hpi). **(D,E)** Cell viability kinetics (%) upon infection (hpi). **(G–I)** HA titer profile (µg/ml) upon infection (hpi). Data are expressed as a mean of two replicates (*n* = 2).

Six different influenza VLPs with distinct valences (i.e. monovalent and pentavalent) and from different groups (A and B) and subtypes (H1 and H3) were produced in insect High Five cells using the baculovirus expression vector system (IC-BEVS). Cell infection and HA protein expression kinetics are shown in Figure 1. Cell concentration and viability profiles follow similar trends in all groups/subtypes and valences studied, except for group B cultures (i.e. culture time extends to 72 h post-infection (hpi) instead of 48 hpi as in other groups/subtypes) (Figures 1A–F). As to protein expression, results suggest that HA titer is group/subtype and valency dependent (Figures 1G–I). For example, while the H1 subtype monovalent culture shows higher HA titers compared to pentavalent culture, the opposite occurs for H3 and B. Overall, the highest HA titer was observed for B pentavalent culture, with peak production reached at 72 hpi.

### Biochemical Characterization of Influenza VLPs

#### Identification of Influenza M1 and HA Proteins

**FIGURE 2:**
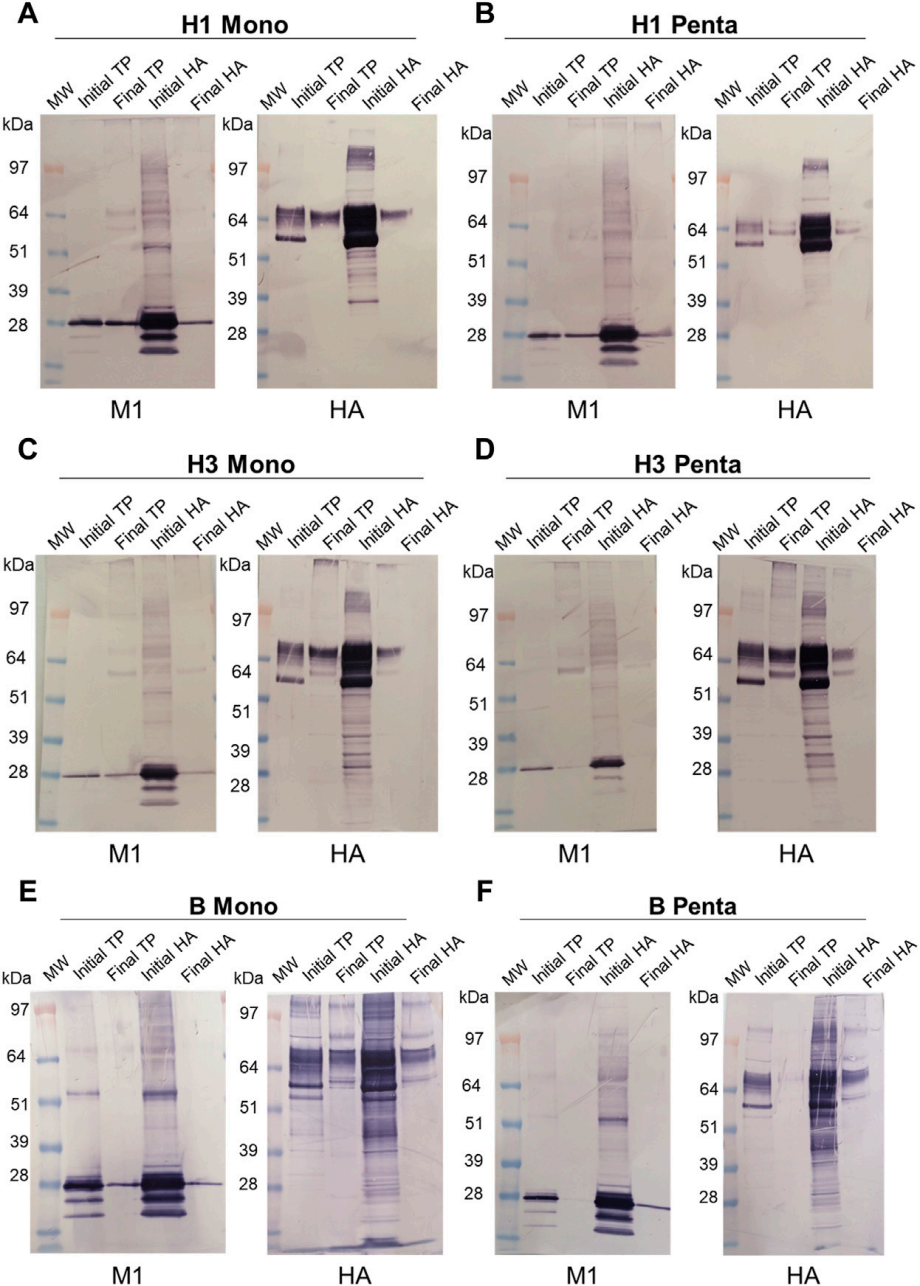
Identification of HA and M1 proteins by Western blot in VLP samples after harvest and after downstream processing (SEC fraction selected). **(A)** H1 Mono VLP, **(B)** H1 Penta VLP, **(C)** H3 Mono VLP, **(D)** H3 Penta VLP, **(E)** B Mono VLP, **(F)** B Penta VLP. MW: molecular weight protein markers; TP: total protein; initial TP: harvest sample loaded by total protein amount; final TP: SEC fraction selected sample loaded by total protein amount; initial HA: harvest sample loaded by HA protein amount; final HA: SEC fraction selected sample loaded by HA protein amount. HA MW = 63 kDa, M1 MW = 28 kDa.

To investigate if both HA and M1 proteins are incorporated in the particles, Western blot analysis was performed at the time of harvest in supernatant samples (initial) and after Downstream processing (DSP), using SEC selected fraction (final) (Figure 2). HA and M1 proteins were specifically identified in both samples, at the expected molecular weight range (HA MW = 63 kDa, M1 MW = 28 kDa), for all the studied VLPs. For B Penta VLP it was only possible to confirm M1 and HA presence in the lanes loaded according to HA content (Figure 2F).

### HA Titers and Process-Related Impurities

**FIGURE 3:**
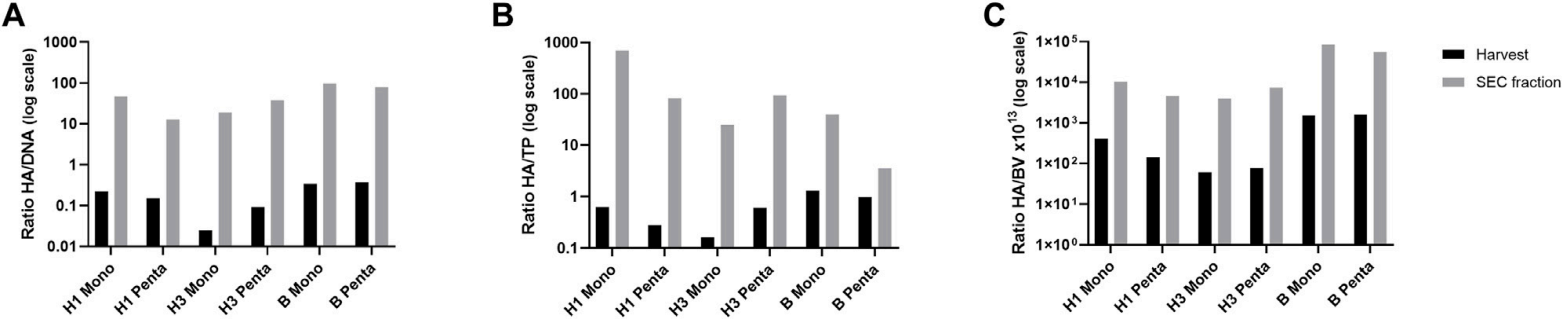
Estimation of HA titers and major process-related impurities. **(A)** HA (µg/ml) to DNA (µg/ml), **(B)** HA (µg/ml) to total protein (mg/ml), and **(C)** HA (µg/ml) to baculovirus concentration (copies/ml × 10^13^) ratios. Log scale is used for visualization purposes. Black bars represent VLP samples after harvest. Grey bars represent VLP samples after downstream processing (SEC fraction selected).

The ratio HA titer to major process-related impurities (i.e., total DNA, total protein, and baculovirus) at harvest and after purification (i.e., SEC fraction) was assessed for all cultures (Figure 3). All ratios are higher for SEC fraction than for harvest, meaning that DSP is increasing HA protein and decreasing impurity content. The HA/DNA ratio (μg/μg) for H1 Mono and H1 Penta VLPs were 0.22 and 0.15 for the harvested samples, respectively (Figure 3A). These values increased to 47.06 and 12.82 after purification. H3 Mono and H3 Penta VLPs have the lowest ratios for the harvest sample, 0.02 and 0.09, respectively. The values increased, after purification, to 19.02 and 38.30. Although the H3 Mono ratio is still one of the lowest, it is not as low as the one obtained for H1 Penta VLP. On the other hand, B VLPs have the highest ratios for both harvest and SEC fractions. B Mono has 0.34 and 97.85 and B Penta 0.37 and 79.31.

In terms of HA/TP ratio (Figure 3B), H1 Mono and H1 Penta VLPs were 0.62 and 0.28 at the harvest sample, respectively. For the SEC fraction samples, the ratios obtained for these VLPs were the highest amongst all the studied particles. The H1 Mono value of 694.46 stands out, but the second highest is 82.33 and refers to H1 Penta VLP. Similar to that observed for DNA, the H3 Mono value was 0.16, the lowest obtained for harvest samples, which slightly increases to 24.69 after purification. H3 Penta starts with 0.61, but in this case, the value of the SEC fraction is 93.53, which is much higher than that for the monovalent VLP. Interestingly, for B type VLPs, although the harvest sample ratios are the highest, 1.29 for B Mono and 0.98 for B Penta, the SEC values are not as high as the ones obtained for other VLPs. B Mono presents a ratio of 39.90 and B Penta has the lowest value of 3.57.

The HA/BV ratios (Figure 3C) can be grouped according to VLP as they differ by approximately 1 log between type/subtype. H1 Mono and H1 Penta values at the harvest were 4.12 × 10^−12^ and 1.43 × 10^−12^, respectively. After purification, the baculovirus content decreased 25 and 33 times, achieving ratio values of 1.05 × 10^−10^ and 4.67 × 10^−11^, respectively. H3 Mono and H3 Penta VLPs ratios are the lowest for the harvest samples with values of 6.03 × 10^−13^ and 7.78 × 10^−13^, respectively. However, the ratio achieved upon purification was the highest, with values of 4.00 × 10^−11^ and 7.40 × 10^−11^, respectively, and baculovirus content decreased by 66 and 95 times. The highest ratios were obtained for B Mono and B Penta VLPs at both harvests, 1.51 × 10^−11^ and 1.64 × 10^−11^, respectively, and SEC fraction samples, 8.56 × 10^−10^ and 5.60 × 10^−10^, respectively. In these cases, the baculovirus content decreased 57 and 34 times.

### Biophysical Characterization of Influenza VLPs

#### Morphology

**FIGURE 4:**
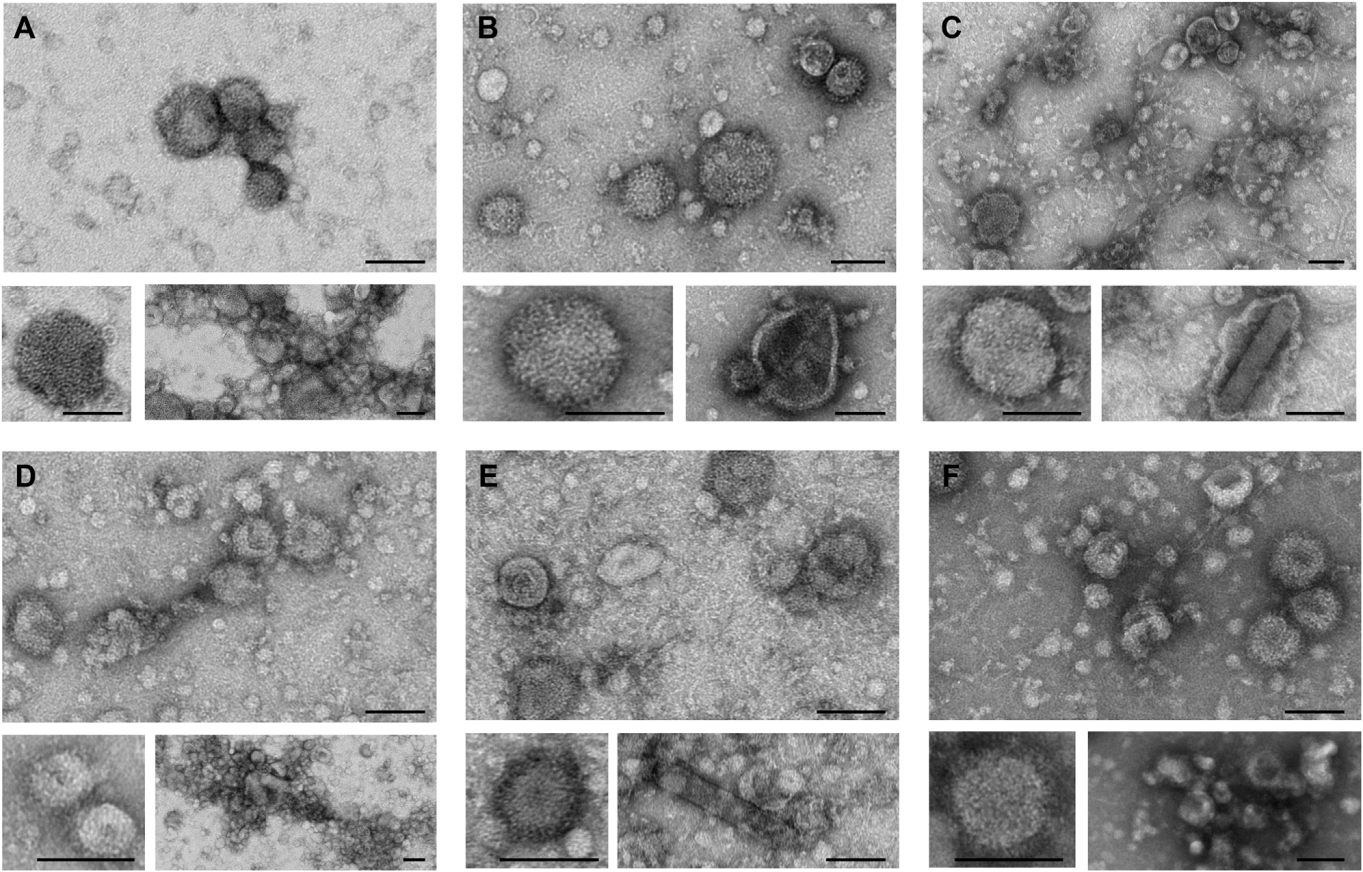
Transmission electron microscopy of purified influenza VLPs. Representative images of TEM analysis depicting VLP size and morphology of purified samples (SEC fraction selected). Top panels show an overview of the analyzed samples; bottom left panels show close up views of individual spherical VLPs presenting the characteristic influenza HA spikes; bottom right panels show the presence of larger and pleomorphic VLPs, aggregates or baculovirus. **(A)** H1 Mono VLP, **(B)** H1 Penta VLP, **(C)** H3 Mono VLP, **(D)** H3 Penta VLP, **(E)** B Mono VLP, **(F)** B Penta VLP. Scale bar, 100 nm.

TEM analysis was performed to assess the integrity and morphology of the six different influenza VLPs, after the complete DSP (Figure 4). Top panels present an overview of each preparation, where the presence of spherical and pleomorphic VLPs can be observed, as well as other types of particles and/or vesicles. It is possible to observe ultrastructural details of VLP membranes by zooming in representative images of each preparation (bottom left panels). All particle images revealed the presence of HA spikes, characteristic of influenza viruses. The bottom right panel images represent the type of impurities observed in each preparation. Besides vesicles and/or exosomes that are visible in all VLP preparations, aggregates can be observed in H1 Mono (Figure 4A), H3 Penta (Figure 4D), and B Penta (Figure 4F) samples. The presence of baculovirus in purified VLP samples is exemplified for H3 Mono (Figure 4C) and B Mono (Figure 4E). Deformed H1 Penta VLPs were also observed (Figure 4B).

### Concentration, Size Distribution, and Surface Charge

**FIGURE 5:**
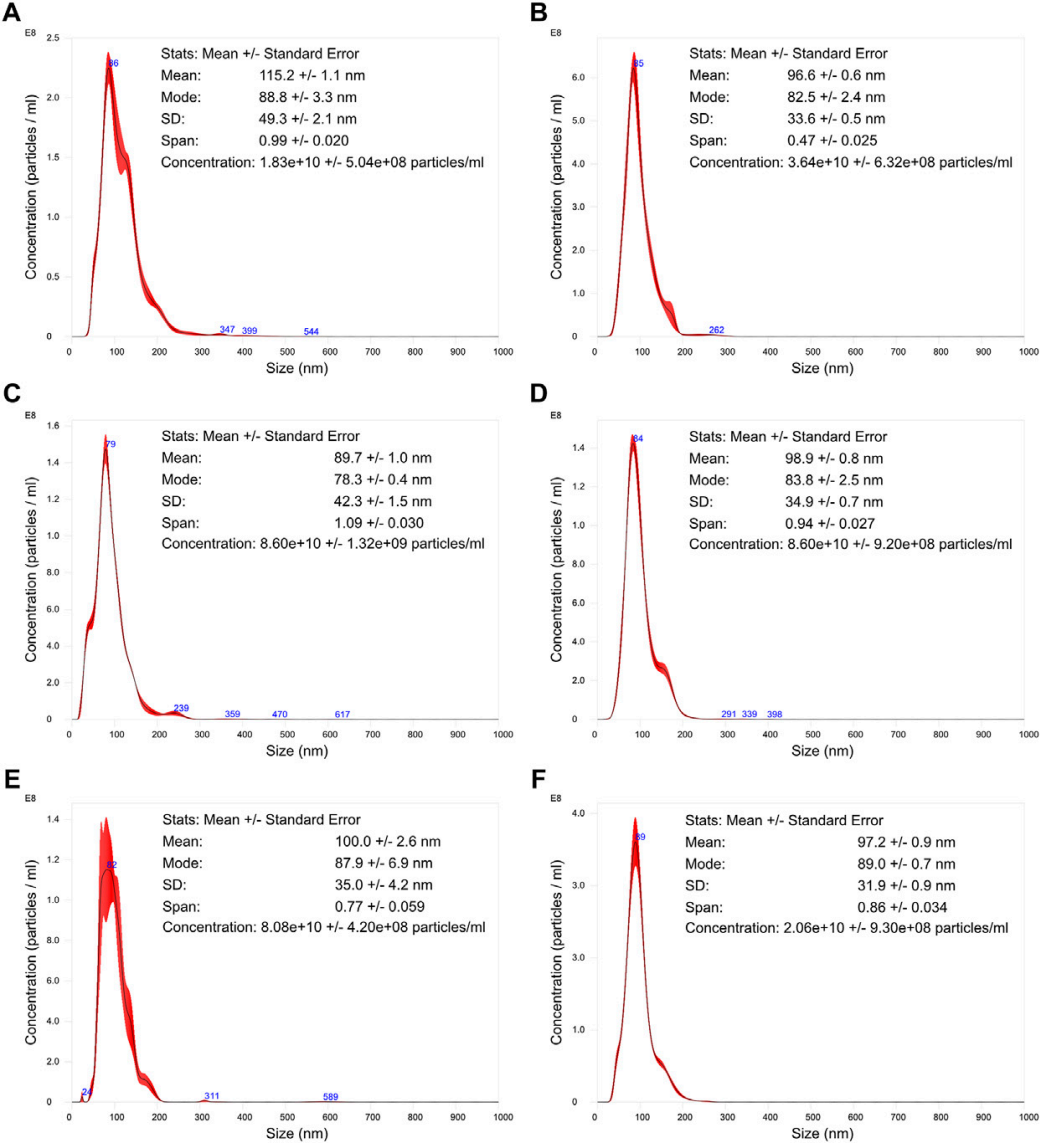
Nanoparticle tracking analysis of influenza VLPs. Particle size distribution analysis of purified samples. The represented distributions are an averaged result of five measurements. The error bars (in red) indicate +/– 1 standard error of the mean. **(A)** H1 Mono VLP, **(B)** H1 Penta VLP, **(C)** H3 Mono VLP, **(D)** H3 Penta VLP, **(E)** B Mono VLP, **(F)** B Penta VLP.

**FIGURE 6:**
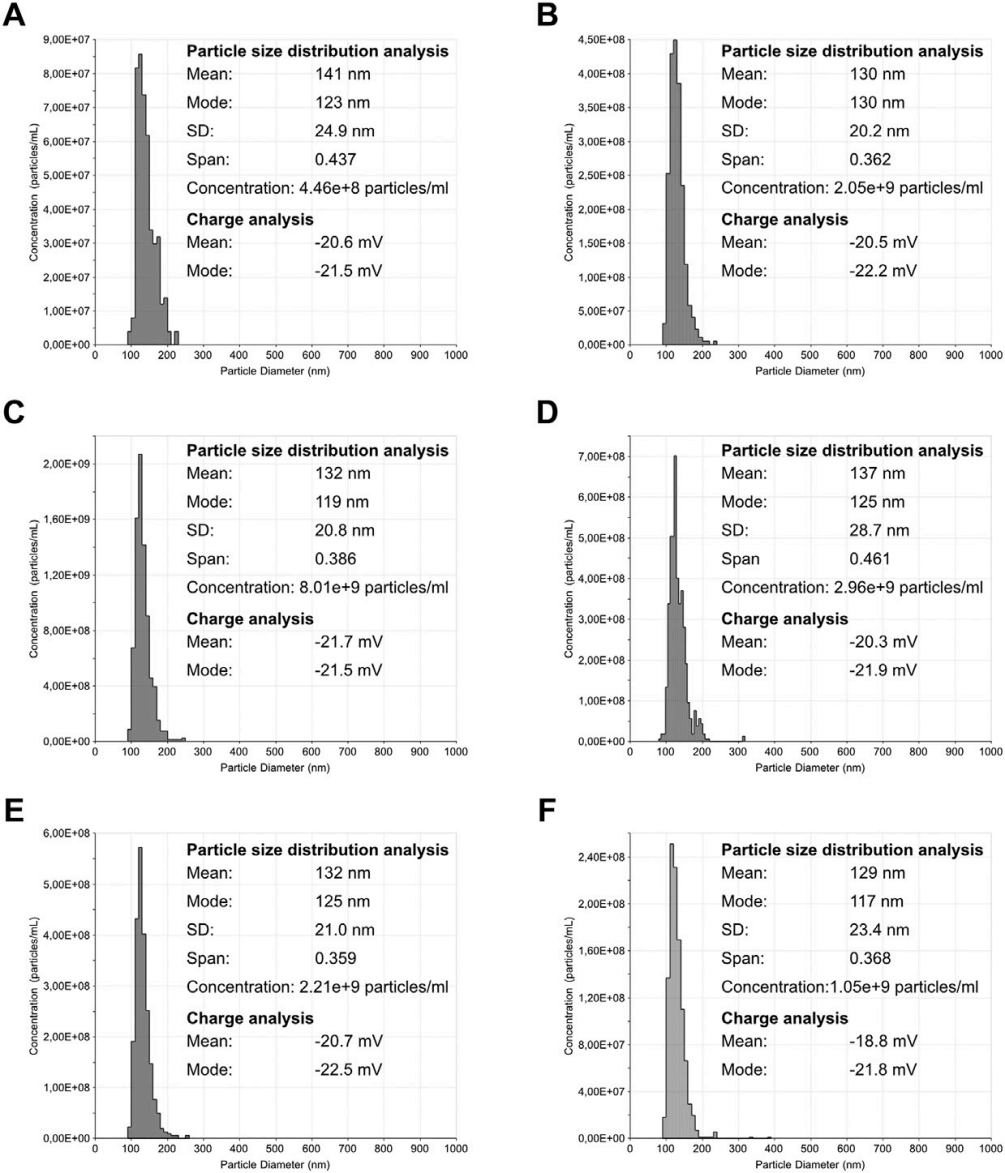
Tunable resistive pulse sensing analysis of influenza VLPs. Particle size distribution analysis of purified samples. The bin size for all histogram was defined as 10 nm. **(A)** H1 Mono VLP, **(B)** H1 Penta VLP, **(C)** H3 Mono VLP, **(D)** H3 Penta VLP, **(E)** B Mono VLP, **(F)** B Penta VLP.

Particle size distribution, concentration, and surface charge were assessed for each VLP purified sample (Figures 5, 6). The particle size distribution determined using TRPS had a mono-modal profile. On the other hand, NTA data suggests a heterogeneous population with three main groups existing: the center group corresponding to a mean value of 79–86 nm and two transitions to lower and higher particle diameters. Mean particle size diameters determined using NTA ranged from 90 to 115 nm, with no visible correlation to monovalent or pentavalent VLPs. A similar observation was obtained with NTA for the mode of the distribution (ranging from 78 to 89 nm). All particle size distributions were positively skewed as their mean was greater than the mode. The same pattern of distribution skewness was observed in the TRPS measurements. The mode and mean values of the particle size distribution obtained with TRPS were higher than the ones obtained with NTA, ranging from 129 to 141 nm for the mean and from 117 to 130 nm for the mode. The polydispersity of the VLP samples was evaluated using a percentile diameter ratio (D90-D10)/D50, usually a named span. The calculated values for span using TRPS were in the lower range (0.359–0.461) in comparison to the ones obtained with NTA (0.47–1.09). No correlation exists between monovalent or pentavalent VLPs and the calculated values of span for TRPS or NTA.

The concentration of total particles determined with NTA was one order of magnitude higher in comparison to the TRPS. The concentration in NTA ranged from 1.83 × 10^10^ to 8.60 × 10^10^ total particles/ml and in TRPS from 4.46 × 10^8^ to 8.01 × 10^9^ total particles/ml.

Zeta potential analysis of the different VLPs ranged from −18.8 to −21.7 mV. Although for H1 Mono and H1 Penta VLP samples the measured zeta potential was very similar (−20.6 and −20.5 mV), the same cannot be observed for the other VLP samples. The pentavalent H3 and B VLPs were less negatively charged, having a zeta potential of −20.3 and −18.8 mV respectively, in comparison with −21.7 mV for H3 Mono and −20.7 mV for B Mono.

### Secondary Structure Analysis and Thermal Stability Evaluation

**FIGURE 7:**
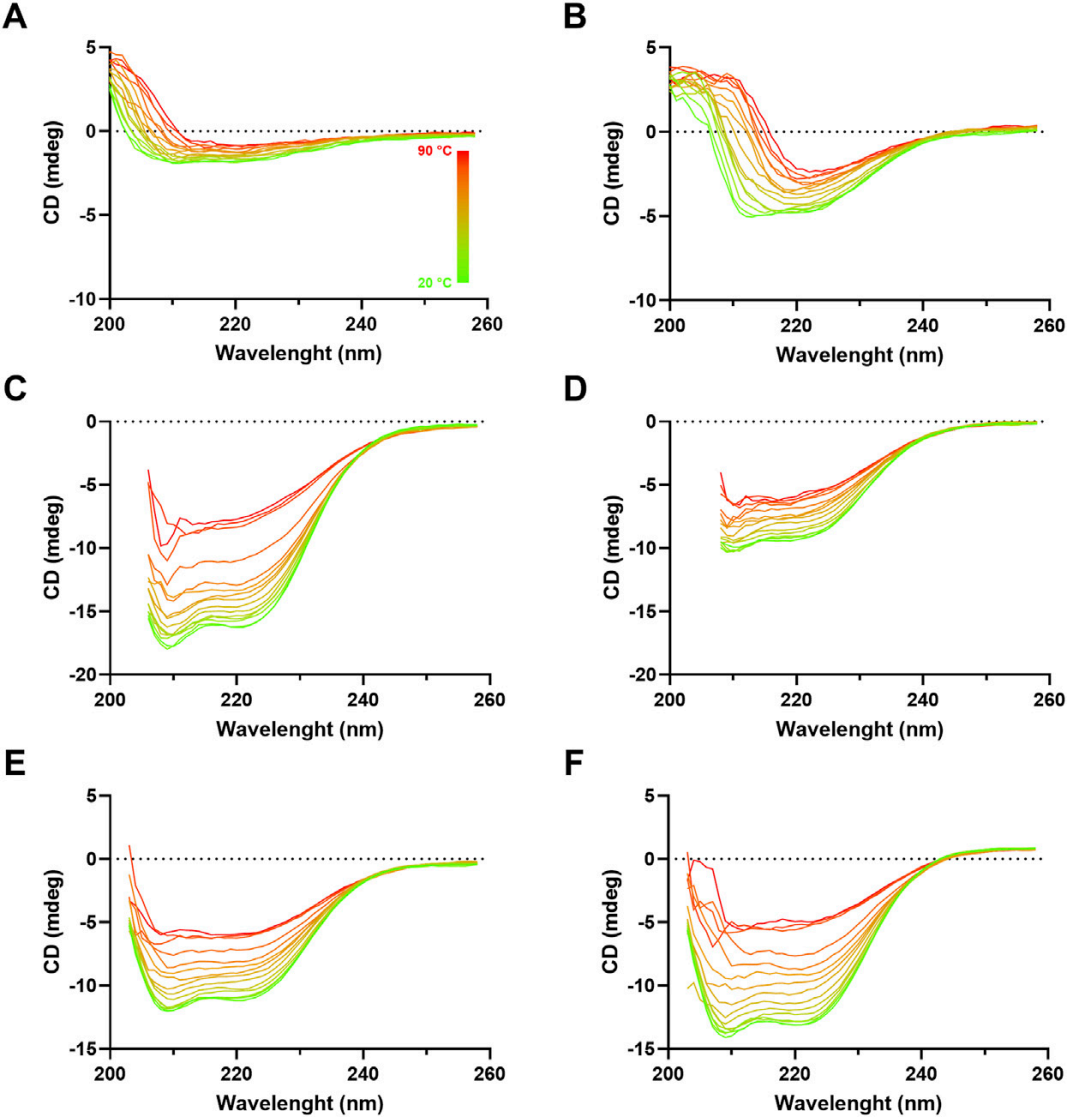
Circular dichroism spectra of influenza VLPs. Far UV (260–195 nm) CD spectra analysis of purified samples (SEC fraction selected) at different temperatures, ranging from 20°C (green) to 90°C (red), pH 7. mdeg: milidegrees. **(A)** H1 Mono VLP, **(B)** H1 Penta VLP, **(C)** H3 Mono VLP, **(D)** H3 Penta VLP, **(E)** B Mono VLP, **(F)** B Penta VLP.

To investigate influenza VLP secondary structure, circular dichroism (CD) analysis of purified VLPs was performed (Figure 7). Although CD spectra at 20°C of the different VLPs present distinct shapes and magnitudes, they display minima near 210 and 222 nm, suggesting the presence of predominantly helix-α secondary structure type. An exception is made for H1 Mono VLP, where magnitude is the lowest and the α-helix signature is not that evident (Figure 7A). As we increase the temperature (from 20°C to 90°C) it is possible to observe a loss of signal, which is indicative of temperature-dependent loss of secondary structure. This signal decrease is more evident for H3 Mono (Figure 7C), B Mono (Figure 7E), and B Penta (Figure 7F) VLPs.

**FIGURE 8:**
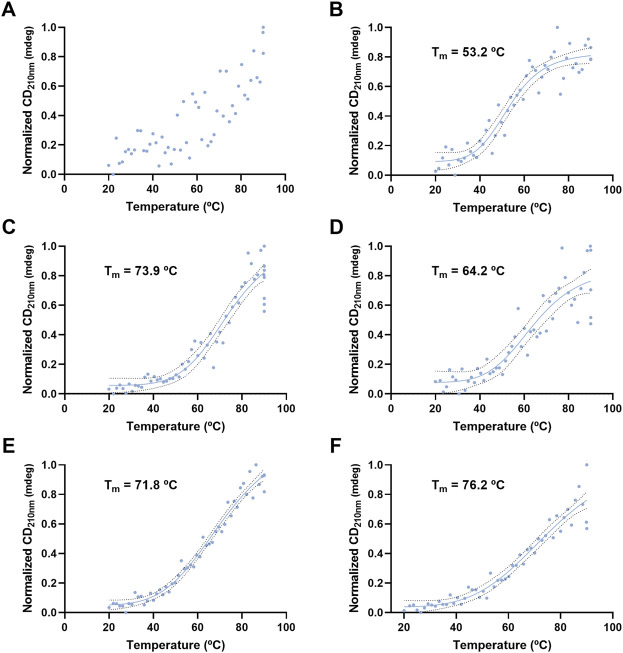
Thermal stress analysis of influenza VLPs using circular dichroism. Temperature ramp for VLP secondary structure analysis during thermal stress. CD signal was followed at 210 nm. Data was normalized (0–1) for plotting. mdeg: milidegrees. **(A)** H1 Mono VLP, **(B)** H1 Penta VLP, **(C)** H3 Mono VLP, **(D)** H3 Penta VLP, **(E)** B Mono VLP, **(F)** B Penta VLP. Indirect measurement of *T*_m_ values was estimated by interpolation applying a sigmoidal regression.

**FIGURE 9:**
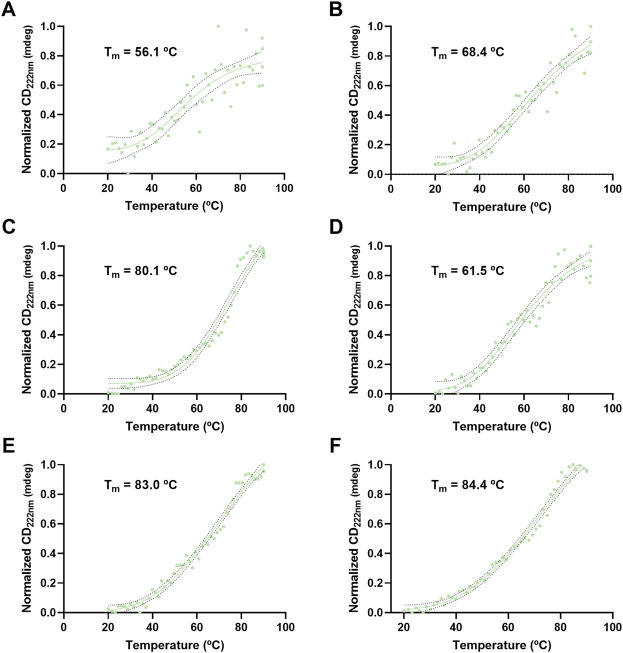
Thermal stress analysis of influenza VLPs using circular dichroism. Temperature ramp for VLP secondary structure analysis during thermal stress. CD signal was followed at 222 nm. Data was normalized (0–1) for plotting. mdeg: milidegrees. **(A)** H1 Mono VLP, **(B)** H1 Penta VLP, **(C)** H3 Mono VLP, **(D)** H3 Penta VLP, **(E)** B Mono VLP, **(F)** B Penta VLP. Indirect measurement of T_m_ values was estimated by interpolation applying a sigmoidal regression.

VLP thermal stability was evaluated by monitoring the CD signal at 210 and 222 nm as a function of temperature (Figures 8, 9). For H1 Mono VLPs (Figure 8A) it is not possible to observe a significant shift in the structure following the 210 nm wavelength. However, we can observe a sigmoidal behavior and a T_m_ of 56.1°C following the 222 nm wavelength (Figure 9A). Considering the 222 nm wavelength, H1 Penta VLPs are more stable than H1 Mono VLPs (ΔT_m_ >12°C) (Figures 9A,B). Contrarily, for the H3 subtype, the monovalent VLPs appear to be more stable than the pentavalent VLPs, irrespective of the wavelength (Figures 8C,D, 9C,D). B VLPs present the highest *T*_m_ at 222 nm, with values of 83.0°C for B Mono (Figure 9E) and 84.4°C for B Penta (Figure 9F). Pentavalent VLP is slightly more stable than the monovalent, for both wavelengths. From the CD signal at 210 nm the obtained values for Tm are 71.8°C for B Mono (Figure 8E) and 76.2°C for B Penta (Figure 8F), respectively.

## Discussion

Influenza VLPs of different groups (A and B), subtypes (H1 and H3), and valences (mono and pentavalent) were produced using the IC-BEVS and characterized biochemically and biophysically. These analyses provide us insights into VLP titer, process-impurities content, morphology, size distribution, surface charge, secondary structure, and thermal stability. VLPs with spherical and pleomorphic morphology were identified, displaying HA on their surface, resembling the influenza virus. Differences were observed for HA titer and DNA, total protein, and baculovirus content across the bioprocess. Although most of the particles present similar secondary structure content and their surface charge is within the same range of values, significant variations in terms of thermal stability were observed.

### VLPs Bioprocess and Biochemical Characterization

The differences in VLP formats (mono or pentavalent), groups (A and B), and subtypes (H1 and H3) may impact upstream and downstream processing stages. Cell concentration and viability profiles followed similar trends in all groups/subtypes and valences studied. Cell viability and HA titer were used to decide the time of harvest. As the expression system used is lytic, we need to consider that cell viability will decrease significantly after infection releasing a considerable amount of host cell impurities. Therefore, it is critical to balance the titer with cell viability as it will impact the downstream processing and product quality. For the case of B VLPs, increasing culture time did not affect cell viability and improved HA titer. No significant differences were observed in terms of impurity content removal when compared to the other VLPs, meaning that it is worth extending cell culture. On the other hand, H3 Mono VLP presented the lowest cell viability at the time of harvest, which was then reflected in terms of impurity content at the initial stage of the purification process, requiring a more robust downstream process. Moreover, the HA titer for this VLP is the lowest observed, meaning that some process optimizations should be taken into consideration (e.g. increase genetic stability of baculovirus and/or protein construct, tailor-made supplementation strategies, perfusion cultures).

We confirmed that all particles incorporated both M1 and respective HA proteins. Biochemical analysis was performed to assess downstream process efficiency in terms of DNA, total protein and baculovirus removal. No evident relation between the subtype, group, or VLP valency was observed. Nevertheless, these analytics can give us insightful information for bioprocess improvement. Further studies to identify which are the host-specific proteins from a baculovirus expression system included in our VLPs will be interesting and can be evaluated by mass spectrometry proteomic analysis (Shaw et al., 2008; Hutchinson et al., 2014; Wang et al., 2017).

### VLP Morphology, Surface Charge and Size Distribution

It is widely reported that influenza viruses are pleomorphic, presenting spherical but also filamentous shapes (Harris et al., 2006; Harris et al., 2013; Calder et al., 2010). In this work, we report TEM imaging of VLPs demonstrating similarity to what is expected for the native virus, in terms of size, morphology and ultrastructural details, namely HA characteristic spikes. These VLPs show a predominantly spherical morphology being also possible to identify other vesicles and baculovirus. These are critical product-related impurities, as their surface properties are usually very similar to the ones of VLPs. Although some reports suggest that these species may have a positive impact on the immune response, there is still a concern from the regulatory authorities on live contaminating viruses or virus genomes in human vaccines due to their potential side effects. In this sense, these impurities should be monitored and quantified, enforcing their removal during the bioprocess (Margine et al., 2012; Thompson et al., 2015; Deng, 2018; Lavado-García et al., 2020). Imaging tools are also useful to identify VLP aggregates and not properly formed particles or evaluate membrane protein density. For example, the H3 Mono and H3 Penta VLPs seem to have a lower HA density in the membrane surface when compared to the other VLPs. This observation is in agreement with the HA titers, which are the lowest observed. Systematic analysis of TEM images can be performed to do a semi-quantification of VLPs and product-related impurities, allowing the monitoring of the process and the optimization of process conditions, e.g., pH or conductivity. Moreover, a deeper characterization of the final product can be performed using cryo-EM (McCraw et al., 2018).

Particle size and size distribution observed by TEM are in agreement with NTA and TRPS measurements. There is no correlation between size or size distribution with the group/subtype of VLPs. The values obtained for the VLP diameter using both techniques are in agreement with the expected size diameter of an influenza virus (80–130 nm) (Tidona and Darai, 2011) and recent reports of VLP size diameter measured by DLS (126 ± 11 nm) (Wu et al., 2010). The difference between the measured size diameters observed for NTA and TRPS can be explained by the different size measurements principle used. NTA determines size distributions and concentrations of particles based on their Brownian motion, calculating the diffusion coefficient of each particle in the dispersant through the Stokes-Einstein equation. The particles’ hydrodynamic diameter is then calculated as a diameter of an equivalent sphere with the same diffusion coefficient (Kim et al., 2019). TRPS on the other hand uses a single dynamically resizable nanopore. When particles cross the pore, there is a change in electrical resistance, producing a pulse in electrical current. This pulse record is then interpreted to access information on geometric particle diameters, particle counts, size distributions, concentrations, and charge (Willmott, 2018).

The observed polydispersity measured through the distribution span reported for both techniques is also different, being on average two times higher in NTA. A closer analysis of the NTA particle size distributions reveals the presence of a secondary population to the left and right of the main population peak, which might not only contribute to the increase in particle dispersion but also for particle counts. TEM imaging reveals the presence of particles with dimensions above and below the VLPs observed. The secondary populations observed in NTA are not evident in TRPS, which might be related to the combination of the nanopore selected and operating parameters. While increasing pore dimensions (through the selection of a wider nanopore membrane, or by increasing the membrane stretch) increases the capability of measuring particles with higher diameters, this also reduces the signal-to-noise ratio of particles at the lower end of detection. The inverse operation will lead to opposite outcomes; lower pore dimensions will provide a reduced capability of measuring particles with higher diameters but will improve the detection of smaller particles.

In this study, and apart from H1 Mono VLPs, NTA yields on average a particle concentration 20 times higher in comparison to TRPS. Further work is still needed to access the reproducibility of the results. These differences are related to the measurement principle of both techniques. However, a recent study (Vogel et al., 2021), where these two sizing techniques were compared, among others, for the measurement of different particle types [synthetic NIST (National Institute of Standards and Technology)-traceable particles, liposomes and exosomes], show that NTA and TRPS can be used to obtain concentration and size distributions comparable within an order of magnitude. In addition to concentration and size measurements, TRPS enables also the measurement of zeta potential. The results obtained are in line with reported values for influenza VLPs (−24 ± 0.2 mV) (Hu et al., 2017). Having access to this surface property can help in the design of the purification process and associated buffers as well as understanding how formulation can impact particle stability.

### VLPs Structure and Thermal Stability

VLP secondary structure was assessed to better understand VLP major components. The presence of a double minima near 210 and 222 nm in the CD spectra suggests that VLP contain a considerable amount of α-helix content, as reported for influenza HA protein (Flanagan and Skehel, 1977). This suggests that the majority of the CD signal observed derives from HA, although we cannot rule out to also be derived from M1 (the VLP scaffold), HCP, or other cellular components. In fact, H1 Mono VLP is the one presenting the lowest CD signal and the highest ratio of HA and impurities, meaning that impurities can be contributing to the α-helix content. There are algorithms available that allow CD signal deconvolution, meaning determination and quantification of each type of secondary structure present in the sample. Here, we evaluated DICHROWEB and CAPITO servers (Whitmore and Wallace, 2008; Wiedemann et al., 2013). Both presented results consistent with the majority of the secondary structure content being α-helix, with no differences among the VLPs studied (data not shown). Nevertheless, the deconvolutions were considered inconclusive as RMSD (root-mean-square deviation) values are higher than the recommended threshold and our data does not fit properly with the data sets available for calibration. Further optimization of the experimental conditions are still needed to improve acquisition parameters, for example, recording CD signal at lower wavelengths and having a reliable analysis. Moreover, the differences observed in thermal stability suggest that CD is a useful tool to evaluate VLP behavior upon changes in process conditions. Interestingly, the thermal shift assay method was also evaluated as a tool to assess stability, but no significant shifts in the structure were observed at the conditions evaluated (data not shown). Using CD, it is also possible to evaluate other temperature ranges, pHs, the effect of cryopreservants, and perform batch-to-batch comparison (Kissmann et al., 2011; Khrustalev et al., 2020).

Hemagglutinin biological activity was confirmed with HA assay. Moreover, a previous study comparing HA assay and biolayer interferometry analysis showed a correlation between both methods, also confirming that these VLPs are also able to bind to sialic acid glycoconjugates, their receptors in cells (Carvalho et al., 2017).

Besides the methods used here, other options and orthogonal tools may be considered, depending on the resources available and specific needs, as reported in Table 1. The methods selected should fit the purpose: in-process monitoring or final product characterization. Bioprocess optimization will require information about titer, particle size, and size distribution, as well as impurity removal. Hemagglutination assay can provide a measurement of HA content during the entire bioprocess. This can also be obtained using orthogonal tools, such as BLI or SPR, presenting several advantages (Nilsson et al., 2010; Carvalho et al., 2017), however at a higher cost of investment. Evaluation of particle size and concentration is also fundamental and requires in-process tools such as NTA, TRPS, or DLS. These methods can give us complementary insights into particles’ characteristics. Particle size and size distribution can be assessed using both NTA and TRPS methods which provide a particle by particle measurement whereas the traditional DLS offers an ensemble measurement. If particle charge is important at this point of the process, TRPS or other zeta potential analyzers should be considered instead. Impurities such as DNA, total protein and, for the expression system used, baculovirus content need to be monitored.

Product characterization implies a set of analytical tools to look at morphology, protein identity, thermal stability, and aggregation propensity, among others. The selection of the analytical method will depend on the depth of VLP characterization. TEM is critical for particle integrity and assessing the presence of HA spikes. It is a gold standard technique for direct particle detection and an immediate overview of the relative amount and shape of the particles. It requires a contrast medium for sample visualization, being that negative staining is the most common. The staining does not penetrate the membrane but the particle is enveloped with the staining compound, which can be challenging when dealing with enveloped VLPs. Moreover, VLP samples can be degraded during sample preparation. More structural information, at the atomic range level, can be obtained with cryo-EM. This technique enables the characterization of the 3D structure of macromolecules, allowing the visualization of viruses and VLPs in their native conformation. It does not require a contrast solution and due to a rapid freezing process sample damage is reduced. However, the costs are higher and the sample preparation is laborious comparing with TEM (Richert-Pöggeler et al., 2019; González-Domínguez et al., 2020). Methods such as CD or DSC allow thermal stability assessment. DSC enables direct characterization of thermal unfolding of biomolecules without the need for reporters. CD follows unfolding transition temperature (Tm) and provides information on secondary structure. However, these methods are optimized for proteins and have limitations, in particular when we are analyzing complex particles, such as VLPs. Although we are characterizing a purified sample, VLPs are complex and heterogeneous entities, and we need to consider not only HA but all the other proteins present. We should bear in mind that like all the spectroscopies if there is heterogeneity all we can observe is an average of the population. Western blot is the traditional method for identity. Higher resolution techniques, such as peptide mapping using MS tools, can give detailed information regarding protein sequence.

The complexity of influenza VLPs (e.g. enveloped particles, multi-protein display) and the expression system (e.g. product and process-related impurities) makes VLP characterization and process monitoring challenging. In this work we (1) demonstrated the feasibility of the methods investigated to characterize the different influenza VLPs produced, and (2) established a criteria grid to aid the selection of the critical and most suitable methods for in-process monitoring and final product characterization, guiding bioprocess optimization. Importantly, the applicability of the methods herein studied can potentially be extended to other VLPs or viruses, produced in IC-BEVS or other virus-based expression systems.

## Data Availability

The raw data supporting the conclusion of this article will be made available by the authors, without undue reservation.
