# Supplementary material for: Bioanalytics for Influenza Virus-Like Particle Characterization and Process Monitoring
Source: Front Bioeng Biotechnol. 2022 Feb 18;10:805176. doi: 10.3389/fbioe.2022.805176 (PMC8894879; doi:10.3389/fbioe.2022.805176)
Supplement: Supplementary file 1 [file DataSheet1.docx]

Supplementary Material

**Table 1**. List of influenza VLPs

| Group | Subtype | Valency | HA gene(s) from strain(s): |
| --- | --- | --- | --- |
| 1 | H1 | Monovalent | A/Brisbane/59/2007 |
|  |  | Multivalent | A/Puerto_Rico/8/1934  A/USSR/90/1977  A/Texas/36/1991  A/New_Caledonia/20/1999  A/Brisbane/59/2007 |
|  | H3 | Monovalent | A/Fujian/411/2002 |
|  |  | Multivalent | A/Hong_Kong/1/1968  A/England/321/77  A/Sichuan/2/1987  A/Johannesburg/33/94  A/Fujian/411/2002 |
| 2 | B | Monovalent | B/Malaysia/2506/2004 |
|  |  | Multivalent | B/Hong Kong/8/1973  B/Victoria/2/87  B/Yamagata/16/1988  B/Jiangsu/10/2003  B/Malaysia/2506/2004 |

*All constructs enclose M1 gene from strain A/California/06/2009(H1N1)*

**Table 2**. List of influenza HA and M1 protein sequences

| Strain | HA protein sequence (Group 1 - Subtype H1) |
| --- | --- |
| A/Puerto Rico/8/1934 | MKANLLVLLCALAAADADTICIGYHANNSTDTVDTVLEKNVTVTHSVNLLEDSHNGKLCRLKGIAPLQLGKCNIAGWLLGNPECDPLLPVRSWSYIVETPNSENGICYPGDFIDYEELREQLSSVSSFERFEIFPKESSWPNHNTNGVTAACSHEGKSSFYRNLLWLTEKEGSYPKLKNSYVNKKGKEVLVLWGIHHPPNSKEQQNLYQNENAYVSVVTSNYNRRFTPEIAERPKVRDQAGRMNYYWTLLKPGDTIIFEANGNLIAPMYAFALSRGFGSGIITSNASMHECNTKCQTPLGAINSSLPYQNIHPVTIGECPKYVRSAKLRMVTGLRNIPSIQSRGLFGAIAGFIEGGWTGMIDGWYGYHHQNEQGSGYAADQKSTQNAINGITNKVNTVIEKMNIQFTAVGKEFNKLEKRMENLNKKVDDGFLDIWTYNAELLVLLENERTLDFHDSNVKNLYEKVKSQLKNNAKEIGNGCFEFYHKCDNECMESVRNGTYDYPKYSEESKLNREKVDGVKLESMGIYQILAIYSTVASSLVLLVSLGAISFWMCSNGSLQCRICI |
| A/USSR/92/1977 | MKAKLLVLLCALSATDADTICIGYHANNSTDTVDTVLEKNVTVTHSVNLLEDSHNGKLCRLKGIAPLQLGKCSIAGWILGNPECESLVSKKSWSYIAETPNSENGTCYPGYFADYEELREQLSSVSSFERFEIFPKERSWPKHNVTRGVTASCSHKGKSSFYRNLLWLTEKNGSYPNLSKSYVNNKEKEVLVLWGVHHPSNIEDQKTIYRKENAYVSVVSSNYNRRFTPEIAERPKVRGQAGRINYYWTLLEPGDTIIFEANGNLIAPWHAFALNRGFGSGIITSNASMDECDTKCQTPQGAINSSLPFQNIHPVTIGECPKYVRSTKLRMVTGLRNIPSIQSRGLFGAIAGFIEGGWTGMIDGWYGYHHQNEQGSGYAADQKSTQNAINGITNKVNSVIEKMNTQFTAVGKEFNKLEKRMENLNKKVDDGFLDIWTYNAELLVLLENERTLDFHDSNVKNLYEKVKSQLKNNAKEIGNGCFEFYHKCNNECMESVKNGTYDYPKYSEESKLNREKIDGVKLESMGVYQILAIYSTVASSLVLLVSLGAISFWMCSNGSLQCRICI |
| A/Texas/36/1991 | MKAKLLVLLCAFTATYADTICIGYHANNSTDTVDTVLEKNVTVTHSVNLLEDSHNGKLCRLKGIAPLQLGNCSVAGWILGNPKCESLFSKESWSYIAETPNPENGTCYPGYFADYEELREQLSSVSSFERFEIFPKESSWPNHTVTKGVTASCSHNGKSSFYRNLLWLTEKNGLYPNLSKSYVNNKEKEVLVLWGVHHPSNIRDQRAIYHTENAYVSVVSSHYSRRFTPEIAKRPKVRDQEGRINYYWTLLEPGDTIIFEANGNLIAPWYAFALSRGFGSGIITSNASMDECDAKCQTPQGAINSSLPFQNVHPVTIGECPKYVRSTKLRMVTGLRNIPSIQSRGLFGAIAGFIEGGWTGMIDGWYGYHHQNEQGSGYAADQKSTQNAINGITNKVNSVIEKMNTQFTAVGKEFNKLERRMENLNKKVDDGFLDIWTYNAELLVLLENGRTLDFHDSNVKNLYEKVKSQLKNNAKEIGNGCFEFYHKCNNECMESVKNGTYDYPKYSEESKLNRGKIDGVKLESMGVYQILAIYSTVASSLVLLVSLGAISFWMCSNGSLQCRICI |
| A/New Caledonia/20/1999 | MKAKLLVLLCTFTATYADTICIGYHANNSTDTVDTVLEKNVTVTHSVNLLEDSHNGKLCLLKGIAPLQLGNCSVAGWILGNPECELLISKESWSYIVETPNPENGTCYPGYFADYEELREQLSSVSSFERFEIFPKESSWPNHTVTGVSASCSHNGKSSFYRNLLWLTGKNGLYPNLSKSYVNNKEKEVLVLWGVHHPPNIGNQRALYHTENAYVSVVSSHYSRRFTPEIAKRPKVRDQEGRINYYWTLLEPGDTIIFEANGNLIAPWYAFALSRGFGSGIITSNAPMDECDAKCQTPQGAINSSLPFQNVHPVTIGECPKYVRSAKLRMVTGLRNIPSIQSRGLFGAIAGFIEGGWTGMVDGWYGYHHQNEQGSGYAADQKSTQNAINGITNKVNSVIEKMNTQFTAVGKEFNKLERRMENLNKKVDDGFLDIWTYNAELLVLLENERTLDFHDSNVKNLYEKVKSQLKNNAKEIGNGCFEFYHKCNNECMESVKNGTYDYPKYSEESKLNREKIDGVKLESMGVYQILAIYSTVASSLVLLVSLGAISFWMCSNGSLQCRICI |
| A/Brisbane/59/2007 | MKVKLLVLLCTFTATYADTICIGYHANNSTDTVDTVLEKNVTVTHSVNLLENSHNGKLCLLKGIAPLQLGNCSVAGWILGNPECELLISKESWSYIVEKPNPENGTCYPGHFADYEELREQLSSVSSFERFEIFPKESSWPNHTVTGVSASCSHNGESSFYRNLLWLTGKNGLYPNLSKSYANNKEKEVLVLWGVHHPPNIGNQKALYHTENAYVSVVSSHYSRKFTPEIAKRPKVRDQEGRINYYWTLLEPGDTIIFEANGNLIAPRYAFALSRGFGSGIINSNAPMDKCDAKCQTPQGAINSSLPFQNVHPVTIGECPKYVRSAKLRMVTGLRNIPSIQSRGLFGAIAGFIEGGWTGMVDGWYGYHHQNEQGSGYAADQKSTQNAINGITNKVNSVIEKMNTQFTAVGKEFNKLERRMENLNKKVDDGFIDIWTYNAELLVLLENERTLDFHDSNVKNLYEKVKSQLKNNAKEIGNGCFEFYHKCNDECMESVKNGTYDYPKYSEESKLNREKIDGVKLESMGVYQILAIYSTVASSLVLLVSLGAISFWMCSNGSLQCRICI |
| Strain | HA protein sequence (Group 1 - Subtype H3) |
| A/Hong Kong/1/1968 | MKTIIALSYIFCLALGQDLPGNDNSTATLCLGHHAVPNGTLVKTITDDQIEVTNATELVQSSSTGKICNNPHRILDGIDCTLIDALLGDPHCDVFQNETWDLFVERSKAFSNCYPYDVPDYASLRSLVASSGTLEFITEGFTWTGVTQNGGSNACKRGPGSGFFSRLNWLTKSGSTYPVLNVTMPNNDNFDKLYIWGVHHPSTNQEQTSLYVQASGRVTVSTRRSQQTIIPNIGSRPWVRGLSSRISIYWTIVKPGDVLVINSNGNLIAPRGYFKMRTGKSSIMRSDAPIDTCISECITPNGSIPNDKPFQNVNKITYGACPKYVKQNTLKLATGMRNVPEKQTRGLFGAIAGFIENGWEGMIDGWYGFRHQNSEGTGQAADLKSTQAAIDQINGKLNRVIEKTNEKFHQIEKEFSEVEGRIQDLEKYVEDTKIDLWSYNAELLVALENQHTIDLTDSEMNKLFEKTRRQLRENAEDMGNGCFKIYHKCDNACIESIRNGTYDHDVYRDEALNNRFQIKGVELKSGYKDWILWISFAISCFLLCVVLLGFIMWACQRGNIRCNICI |
| A/England/321/77 | MKTIIALSYIFCQVLAQNLPGNDNSTATLCLGHHAVPNGTLVKTITNDQIEVTNATELVQSSSTGRICDSPHRILDGKNCTLIDALLGDPHCDGFQNEKWDLFVERSKAFSNCYPYDVPDYASLRSLVASSGTLEFINEGFNWTGVTQNGGSYACKRGPDNSFFSRLNWLYKSESTYPVLNVTMPNNDNFDKLYIWGVHHPSTDKEQTKLYVQASGRVTVSTKRSQQTIIPNVGSRPWVRGLSSRISIYWTIVKPGDILLINSNGNLIAPRGYFKIRTGKSSIMRSDAPIGTCSSECITPNGSIPNDKPFQNVNKITYGACPKYVKQNTLKLATGMRNVPEKQTRGIFGAIAGFIENGWEGMIDGWYGFRHQNSEGTGQAADLKSTQAAIDQINGKLNRVIEKTNEKFHQIEKEFSEVEGRIQDLEKYVEDTKIDLWSYNAELLVALENQHTIDLTDSEMNKLFEKTRRQLRENAEDMGNGCFKIYHKCDNACIGSIRNGTYDHDVYRDEALNNRFQIKGVELKSGYKDWILWISFAISCFLLCVVLLGFIMWACQKGNIRCNICI |
| A/Sichuan/2/1987 | MKTIIALSYIFCLVFAQKLPGNDNSTATLCLGHHAVPNGTLVKTITNDQIEVTNATELVQSSSTGRICDSPHRILDGKNCTLIDALLGDPHCDGFQNEKWDLFVERSKAYSNCYPYDVPDYASLRSLVASSGTLEFINEDFNWTGVTQSGGSYACKRGSVNSFFSRLNWLHKSEYKYPALNVTMPNNGKFDKLYIWGVHHPITDREQTNLYVRASGRVTVSTKRSQQTVIPNIGSRPWVRGLSSRISIYWTIVKPGDILLINSTGNLIAPRGYFKIRTGKSSIMRSDAPIGTCSSECITPNGSIPNDKPFQNVNKITYGACPRYVKQNTLKLATGMRNVPEKQTRGIFGAIAGFIENGWEGMVDGWYGFRHQNSEGTGQAADLKSTQAAIDQINGKLNRLIEKTNEKFHQIEKEFSEVEGRIQDLEKYVEDTKIDLWSYNAELLVALENQHTIDLTDSEMNKLFEKTRRQLRENAEDMGNGCFKIYHKCDNACIGSIRNGTYDHDVYRDEALNNRFQIKGVELKSGYKDWILWISFAISCFLLCVVLLGFIMWACQKGNIRCNICI |
| A/Johannesburg/33/94 | MKTIIALSYILCLVFAQKLPGNDNSTATLCLGHHAVPNGTLVKTITNDQIEVTNATELVQSSPTGRICDSPHRILDGKNCTLIDALLGDPHCDGFQNKEWDLFVERSKAYSNCYPYDVPDYASLRSLVASSGTLEFINENFNWTGVAQDGKSYACKRGSVNSFFSRLNWLHKLEYKYPALNVTMPNNGKFDKLYIWGVHHPSTDSDQTSLYVRASGRVTVSTKRSQQTVIPDIGYRPWVRGQSSRIGIYWTIVKPGDILLINSTGNLIAPRGYFKIRNGKSSIMRSDAPIGNCSSECITPNGSIPNDKPFQNVNRITYGACPRYVKQNTLKLATGMRNVPEKQTRGIFGAIAGFIENGWEGMVDGWYGFRHQNSEGTGQAADLKSTQAAIDQINGKLNRLVEKTNEKFHQIEKEFSEVEGRIQDLEKYVEDTKIDLWSYNAELLVALENQHTIDLTDSEMNKLFERTRKQLRENAEDMGNGCFKIYHKCDNACIGSIRNGTYDHDVYRDEALNNRFQIKGVELKSGYKDWILWISFAISCFLLCVVLLGFIMWACQKGNIRCNICI |
| A/Fujian/411/2002 | MKTIIALSYILCLVFSQKLPGNDNSTATLCLGHHAVPNGTIVKTITNDQIEVTNATELVQSSSTGGICDSPHQILDGENCTLIDALLGDPQCDGFQNKKWDLFVERSKAYSNCYPYDVPDYASLRSLVASSGTLEFNNESFNWAGVTQNGTSSACKRRSNKSFFSRLNWLTQLKYKYPALNVTMPNNEKFDKLYIWGVHHPVTDSDQISLYAQASGRITVSTKRSQQTVIPNIGYRPRVRDISSRISIYWTIVKPGDILLINSTGNLIAPRGYFKIRSGKSSIMRSDAPIGKCNSECITPNGSIPNDKPFQNVNRITYGACPRYVKQNTLKLATGMRNVPEKQTRGIFGAIAGFIENGWEGMVDGWYGFRHQNSEGTGQAADLKSTQAAINQINGKLNRLIGKTNEKFHQIEKEFSEVEGRIQDLEKYVEDTKIDLWSYNAELLVALENQHTIDLTDSEMNKLFERTKKQLRENAEDMGNGCFKIYHKCDNACIESIRNGTYDHDVYRDEALNNRFQIKGVELKSGYKDWILWISFAISCFLLCVALLGFIMWACQKGNIRCNICI |

| Strain | HA protein sequence (Group 2 – Subtype B) |
| --- | --- |
| B/Hong Kong/8/1973 | MKAIIVLLMVVTSNADRICTGITSSNSPHVVKTATQGEVNVTGVIPLTTTPTKSHFANLKGTQTRGKLCPNCLNCTDLDVALGRPKCMGTIPSAKASILHEVKPVTSGCFPIMHDRTKIRQLPNLLRGYENIRLSARNVTNAETAPGGPYIVGTSGSCPNVTNGNGFFATMAWAVPKNKTATNPLTVEVPYICTKGEDQITVWGFHSDDETQMVKLYGDSKPQKFTSSANGVTTHYVSQIGGFPNQAEDEGLPQSGRIVVDYMVQKPGKTGTIAYQRGVLLPQKVWCASGRSKVIKGSLPLIGEADCLHEKYGGLNKSKPYYTGEHAKAIGNCPIWVKTPLKLANGTKYRPPAKLLKERGFFGAIAGFLEGGWEGMIAGWHGYTSHGAHGVAVAADLKSTQEAINKITKNLNSLSELEVKNLQRLSGAMDELHNEILELDEKVDDLRADTISSQIELAVLLSNEGIINSEDEHLLALERKLKKMLGPSAVDIGNGCFETKHKCNQTCLDRIAAGTFNAGEFSLPTFDSLNITAASLNDDGLDNHTILLYYSTAASSLAVTLMIAIFIVYMVSRDNVSCSICL |
| B/Victoria/2/87 | MKAIIVLLMVVTSNADRICTGITSSNSPHVVKTATQGEVNVTGVIPLTTTPTKSHFANLKGTKTRGKLCPKCLNCTDLDVALGRPKCTGTIPSAKASILHEVKPVTSGCFPIMHDRTKIRQLPNLLRGYEHIRLSTHNVINAETAPGGPYKVGTSGSCPNVTNGNGFFATMAWAVPKNDNNKTATNPLTVEVPYICTEGEDQITVWGFHSDNEAQMVKLYGDSKPQKFTSSANGVTTHYVSQIGGFPNQAEDGGLPQSGRIVVDYMVQKSGKTGTITYQRGILLPQKVWCASGRSKVIKGSLPLIGEADCLHEKYGGLNKSKPYYTGEHAKAIGNCPIWVKTPLKLANGTKYRPPAKLLKEKGFFGAIAGFLEGGWEGMIAGWHGYTSHGAHGVAVAADLKSTQEAINKITKNLNSLSELEVKNLQRLSGAMDELHNKILELDEKVDDLRADTISSQIELAVLLSNEGIINSEDEHLLALERKLKKMLGPSAVEIGNGCFETKHKCNQTCLDRIAAGTFNAGEFSLPTFDSLNITAASLNDDGLDNHTILLYYSTAASSLAVTLMIAIFIVYMVSRDNVSCSICL |
| B/Yamagata/16/1988 | MKAIIVLLMVVTSNADRICTGITSSNSPHVVKTATQGEVNVTGVIPLTTTPTKSHFANLKGTKTRGKLCPNCLNCTDLDVALGRPMCMGTIPSAKASILHEVRPVTSGCFPIMHDRTKIRQLPNLLRGYENIRLSTHNVINAERAPGGPYRLGTSGSCPNVTSRNGFFATMAWAVPRDNKTATNPLTVEVPYICTKGEDQITVWGFHSDDKTQMKNLYGDSNPQKFTSSANGVTTHYVSQIGDFPNQTEDGGLPQSGRIVVDYMVQKPGKTGTIVYQRGVLLPQKVWCASGRSKVIKGSLPLIGEADCLHEKYGGLNKSKPYYTGEHAKAIGNCPIWVKTPLKLANGTKYRPPAKLLKERGFFGAIAGFLEGGWEGMIAGWHGYTSHGAHGVAVAADLKSTQEAINKITKNLNSLSELEVKNLQRLSGAMDELHNEILELDEKVDDLRADTISSQIELAVLLSNEGIINSEDEHLLALERKLKKMLGPSAVDIGNGCFETKHKCNQTCLDRIAAGTFNAGEFSLPTFDSLNITAASLNDDGLDNHTILLYYSTAASSLAVTLMIAIFIVYMVSRDNVSCSICL |
| B/Jiangsu/10/2003 | MKAIIVLLMVVTSNADRICTGITSSNSPHVVKTATQGEVNVTGVIPLTTTPTKSYFANLKGTRTRGKLCPDCLNCTDLDVALGRPMCVGTTPSAKASILHEVRPVTSGCFPIMHDRTKIRQLPNLLRGYENIRLSTQNVIDAENAPGGPYRLGTSRSCPNATSKSGFFATMAWAVPKDNNKNATNPLTVEVPYVCTEGEDQITVWGFHSDNKTQMKNLYGDSNPQKFTSSANGVTTHYVSQIGGFPAQTEDEGLPQSGRIVVDYMVQKPRKTGTIVYQRGVLLPQKVWCASGRSKVIKGSLPLIGEADCLHEKYGGLNKSKPYYTGEHAKAIGNCPIWVKTPLKLANGTKYRPPAKLLKERGFFGAIAGFLEGGWEGMIAGWHGYTSHGAHGVAVAADLKSTQEAINKITKNLNSLSELEVKNLQRLSGAMDELHNEILELDEKVDDLRADTISSQIELAVLLSNEGIINSEDEHLLALERKLKKMLGPSAVDIGNGCFETKHKCNQTCLDRIAAGTFNAGEFSLPTFDSLNITAASLNDDGLDNHTILLYYSTAASSLAVTLMLAIFIVYMVSRDNVSCSICL |
| B/Malaysia/2506/2004 | MKAIIVLLMVVTSNADRICTGITSSNSPHVVKTATQGEVNVTGVIPLTTTPTKSHFANLKGTETRGKLCPKCLNCTDLDVALGRPKCTGNIPSARVSILHEVRPVTSGCFPIMHDRTKIRQLPNLLRGYEHIRLSTHNVINAENAPGGPYKIGTSGSCPNVTNGNGFFATMAWAVPKNDNNKTATNSLTIEVPYICTEGEDQITVWGFHSDNEIQMAKLYGDSKPQKFTSSANGVTTHYVSQIGGFPNQTEDGGLPQSGRIVVDYMVQKSGKTGTITYQRGILLPQKVWCASGRSKVIKGSLPLIGEADCLHEKYGGLNKSKPYYTGEHAKAIGNCPIWVKTPLKLANGTKYRPPAKLLKERGFFGAIAGFLEGGWEGMIAGWHGYTSHGAHGVAVAADLKSTQEAINKITKNLNSLSELEVKNLQRLSGAMDELHNEILELDEKVDDLRADTISSQIELAVLLSNEGIINSEDEHLLALERKLKKMLGPSAVEIGNGCFETKHKCNQTCLDRIAAGTFDAGEFSLPTFDSLNITAASLNDDGLDNHTILLYYSTAASSLAVTLMIAIFVVYMVSRDNVSCSICL |

| Strain | M1 protein sequence (Group 1 - Subtype H1) |
| --- | --- |
| A/California/06/2009(H1N1) | MSLLTEVETYVLSIIPSGPLKAEIAQRLESVFAGKNTDLEALMEWLKTRPILSPLTKGILGFVFTLTVPSERGLQRRRFVQNALNGNGDPNNMDRAVKLYKKLKREITFHGAKEVSLSYSTGALASCMGLIYNRMGTVTTEAAFGLVCATCEQIADSQHRSHRQMATTTNPLIRHENRMVLASTTAKAMEQMAGSSEQAAEAMEVANQTRQMVHAMRTIGTHPSSSAGLKDDLLENLQAYQKRMGVQMQRFK |

**Table 3**. List of primary and secondary antibodies used for western blot analysis

| **Protein identification** | **Antibody name** | **Dilution** | **Reference** |
| --- | --- | --- | --- |
|  | **Primary antibody** |  |  |
| M1 | anti-influenza A virus M1 goat polyclonal antibody | 1:2000 | Abcam ab20910 |
| H1 Mono and H1 Penta | mouse anti-HA FR-494 | 1:2000 | International Reagent Resource (IRR) |
| H3 Mono and H3 Penta | a mixture of sheep anti-HA Nanchang and anti-HA Johannesburg | 1:1000 | NIBSC: 97/612 and NIBSC: 95/524 |
| B Mono and B Penta | a mixture of sheep anti-B Hong Kong and anti-B VICTORIA | 1:1000 | NIBSC: 77/568 and NIBSC: 88/676 |
|  | **Secondary antibody** |  |  |
| M1 | Anti-Goat IgG−Alkaline Phosphatase antibody produced in rabbit | 1:2000 | Sigma, Ref.: A4187 |
| H1 Mono and H1 Penta | Anti-Mouse IgG−Alkaline Phosphatase antibody produced in goat | 1:5000 | Sigma, Ref.: A3438 |
| H3 Mono and H3 Penta | Anti-sheep IgG−Alkaline Phosphatase antibody produced in donkey | 1:5000 | Abcam, Cat# ab6901 |
| B Mono and B Penta | Anti-sheep IgG−Alkaline Phosphatase antibody produced in donkey | 1:5000 | Abcam, Cat# ab6901 |
